# Optimizing dose-schedule regimens with bayesian adaptive designs: opportunities and challenges

**DOI:** 10.3389/fphar.2023.1261312

**Published:** 2023-11-23

**Authors:** Xin Chen, Ruyue He, Xinyi Chen, Liyun Jiang, Fei Wang

**Affiliations:** Research Center of Biostatistics and Computational Pharmacy, China Pharmaceutical University, Nanjing, China

**Keywords:** dose-schedule regimen, adaptive design, dosage optimization, bayesian method, early phase clinical trial

## Abstract

Due to the small sample sizes in early-phase clinical trials, the toxicity and efficacy profiles of the dose-schedule regimens determined for subsequent trials may not be well established. The recent development of novel anti-tumor treatments and combination therapies further complicates the problem. Therefore, there is an increasing recognition of the essential place of optimizing dose-schedule regimens, and new strategies are now urgently needed. Bayesian adaptive designs provide a potentially effective way to evaluate several doses and schedules simultaneously in a single clinical trial with higher efficiency, but real-world implementation examples of such adaptive designs are still few. In this paper, we cover the critical factors associated with dose-schedule optimization and review the related innovative Bayesian adaptive designs. The assumptions, characteristics, limitations, and application scenarios of those designs are introduced. The review also summarizes some unresolved issues and future research opportunities for dose-schedule optimization.

## 1 Introduction

For a long time, dose-finding trials for anti-tumor drugs aim to identify the maximum tolerated dose (MTD), and the MTD, or the next lower dose, will generally be administered in subsequent clinical trials without further optimization. This more-is-better paradigm was originally developed for cytotoxic drugs and is based on the assumption that both efficacy and toxicity increase monotonically with the dose. Thus, MTD is naturally deemed as the most efficacious dose among all safe doses. However, this assumption may not hold for novel molecularly targeted agents (MTAs) and immunotherapies which have much wider therapeutic indices (Bedard et al., 2020; Araujo et al., 2021). In these cases, doses below the MTD may have similar efficacy to the MTD but with fewer toxicities. Sometimes, the MTD even cannot be determined as no dose-limiting toxicity (DLT) is observed. This means the traditional MTD-finding trial design may not be optimal, and comprehensive evaluations for safety, efficacy, dose-response relationships, pharmacokinetic (PK) and pharmacodynamic (PD) characteristics should be incorporated in dose-finding trials. On the other hand, a substantial portion of early phase oncology clinical trials does not fully take into account the effects of dosing schedules (i.e., the interval between doses and duration of treatment) on safety and efficacy, which is not desirable now as patients can often receive those targeted therapies for much longer periods. Therefore, it is necessary to optimize dose-schedule regimens for novel anti-tumor drugs at the stage of clinical development.

There is an increasing recognition of the essential place of optimizing dose-schedule regimens. In 2021, the U.S. Food and Drug Administration (FDA) Oncology Center of Excellence initiated *Project Optimus* to reform the dosage optimization and dosage selection paradigm in oncology drug development (Shah et al., 2021). Its mission is to ensure that doses of anti-tumor drugs are optimized to maximize efficacy as well as safety and tolerability. In January 2023, the FDA issued a draft guidance on optimizing the dosage (refers to the dose and schedule) for the treatment of oncologic diseases (FDA, 2023), encouraging sponsors to plan the drug development programs such that identification of the optimal dosages can occur prior to or concurrently with the establishment of the drug’s safety and effectiveness. We focus on dose-schedule (dosage) optimization in this review, and dose optimization, which may be more commonly seen, refers to optimize the quantity of the drug based on the same schedule here.

Optimizing dose-schedule regimens aims to minimize toxicity while delivering the desired therapeutic effect. It is of great importance in terms of increasing medication compliance, reducing side effects, improving the quality of life, and ultimately, maximizing the benefit-risk ratio for cancer patients. An optimized dose-schedule regimen can also provide an opportunity for patients with poor performance status to receive treatment, as clinicians may be reluctant to treat them with a regimen that does not have a good tolerability and safety profile.

However, due to the small sample sizes in early-phase clinical trials, the toxicity and efficacy profiles of the dose-schedule regimens determined for subsequent trials may not be well established. There are some examples of drugs whose doses or schedules were modified for safety or tolerability after approval (Shah et al., 2021). New strategies for optimizing dose-schedules in early-phase clinical trials are now urgently needed. Bayesian adaptive designs provide a potentially effective way to evaluate several doses and schedules simultaneously in a single clinical trial with higher efficiency. For example, with a rational Bayesian model and prior settings, we can obtain more efficient estimates for toxicity and efficacy. In addition, some Bayesian designs allow borrowing information across different populations or across different dose-schedules, which may increase the probabilities of selecting the optimal dose-schedule and identifying inadmissible dose-schedules. They may also be useful in saving sample size and shortening trial duration. However, real-world implementation examples of such adaptive designs are still few. In this paper, we cover the critical factors associated with dose-schedule optimization and review the related innovative Bayesian adaptive designs. The assumptions, characteristics, limitations, and application scenarios of those designs are introduced. The review also summarizes some unresolved issues and future research opportunities for dose-schedule optimization.

## 2 Optimization: what factors are critical?

**FIGURE 1:**
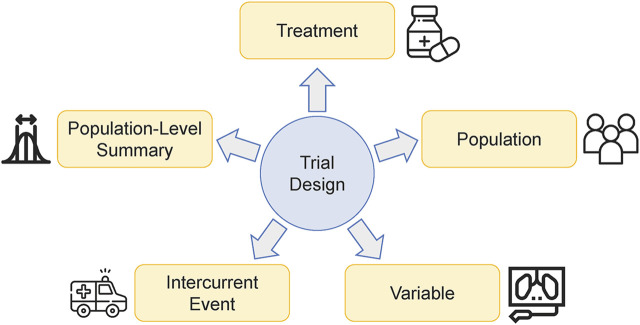
Critical factors that affect the strategy of optimizing dose-schedule regimens can be categorized into the five attributes of the estimand framework.

There are a lot of factors that may shape the strategy of optimizing dose-schedule regimens. The authors found that a clear way to categorize them is to follow the estimand framework (Figure 1), which provides a precise definition of the treatment effects (Akacha and Kothny, 2017). According to ICH E9 (R1), five major attributes are used to construct the estimand, including ‘treatment’, ‘population’, ‘variable’, ‘intercurrent event’, and ‘population-level summary’ (ICH, 2019). In this section, we will go into detail about how those factors affecting dose-schedule optimization are categorized into these five attributes. We believe that familiarity with the estimand framework is of profound significance for designing a dose-schedule optimization trial.

### 2.1 Treatment

The treatment condition should be well-defined in a dose-schedule optimization trial. Candidate doses and schedules can be determined based on preclinical data, clinical data, or other data from compounds in the same drug class. The mechanism of action (MOA) for the investigational treatment induces its specific dose-toxicity and dose-efficacy curves, calling for adopting appropriate designs. For example, those traditional MTD-finding designs such as 3 + 3 design (Storer, 1989) and continual reassessment method (O'Quigley et al., 1990), may be suitable for cytotoxic drugs. If the chronic and cumulative toxicities are of concern, we can further consider determining the maximum tolerated schedule (MTS). For immunotherapies that potentially result in lower grade but persistent symptomatic toxicities, there do exist requirements to optimize both doses and schedules as a lower dose or a longer dosing interval may have similar efficacy to that of the MTD/MTS. The potential orderings of schedules regarding toxicity and efficacy can also greatly affect the choice of trial designs. As is customary in the literature, we refer to ordered/unordered schedules that can be anticipated in the planning stage as the ‘nested/non-nested schedules’. For example, it is reasonable to assume a 14 days on/6 days off schedule is more toxic than a 7 days on/3 days off schedule. So, these two schedules are nested, and it may not be appropriate to randomly assign patients to these two schedules when there are great uncertainties regarding toxicity. An example of non-nested schedules is that Schedule A and B are once every 4 weeks and once every week respectively, given the same total dose. The former schedule may have a higher short-term drug exposure, while the latter has more drug administrations. Therefore, the order of efficacy or toxicity of these two schedules is unclear in the planning stage. If the investigational treatment is a combination of several interventions administered concurrently, whether optimizing the dose-schedule regimens for one or all interventions requires careful consideration.

### 2.2 Population

The identification of patient populations has become increasingly important with the development of targeted therapies. Patient factors (e.g., age and performance status, organ function, previous therapies, histopathology patterns or biomarker expressions) may give rise to different sensitivities to drugs. For example, in the dose-expansion trial of trastuzumab deruxtecan, investigators identified doses for clinical use as 5.4 mg/kg q3w and 6.4 mg/kg q3w for HER2+ breast cancer and HER2+ gastric/gastroesophageal junction adenocarcinoma, respectively (Shitara et al., 2019; Tamura et al., 2019). Therefore, the optimal dose-schedule regimen may not be the same across subpopulations (e.g., different indications, subgroups, or principal stratums defined by the occurrence of a specific intercurrent event). Innovative trial designs, represented by the basket design, make it possible to evaluate one targeted therapy for multiple subpopulations simultaneously in a single trial (Park et al., 2020; Hobbs et al., 2022). Bayesian adaptive designs with information borrowing across subpopulations have the potential to improve the efficiency of clinical trials (Su et al., 2022).

### 2.3 Variable

The variables, or more commonly used in the context of clinical trials, the endpoints, should be pre-specified based on the specific clinical questions. The primary endpoints should be able to support the overall goal of determining a dosage that is safe and effective and does not result in unnecessary toxicities. In a dose-schedule optimization trial, the endpoints to be collected can be considered based on the following aspects.

The first is toxicity, which is usually the primary concern in traditional dose-finding designs for cytotoxic drugs. Ensuring the safety of current and subsequent subjects is one eternal theme for clinical trials. For some anti-tumor therapies, collecting and evaluating chronic, cumulative and low-grade symptomatic toxicities, not just DLTs, may sometimes need to be considered.

The second to be concerned is efficacy. Traditional phase I dose-finding trials for anti-tumor drugs are often based solely on toxicity with a small sample size, ignoring efficacy when selecting doses for future study or clinical practice (Yan et al., 2018). This more-is-better paradigm cannot fully characterize the benefit-risk ratio for cancer patients. Therefore, for recently proposed dosage optimization designs, the most distinctive feature is that they incorporate both efficacy and toxicity endpoints. Due to time constraints, short-term endpoints, such as tumor response or some biomarker expression levels, are commonly used to reflect short-term benefits for patients.

Except for toxicity and efficacy endpoints, collecting and analyzing pharmacokinetic (PK) and pharmacodynamic (PD) data is becoming increasingly important for dosage selection with the development of quantitative pharmacology. In recent years, model-informed drug development (MIDD) has been involved in the determination of dose-schedule regimens for several monoclonal antibody immune checkpoint inhibitors (Peer et al., 2020). An integrated PK/PD analysis approach may help to interpret early clinical data. It may also be beneficial to leverage data from other compounds in the same drug class.

Last but not least, although there are now few explicit practical uses, patient-reported outcomes (PRO) should be considered to enhance the assessment of the benefit-risk ratio in early-phase clinical trials. Collecting and analyzing PROs can provide a systematic and quantitative assessment of symptomatic adverse events and the quality of life, consistent with the concept of patient-focused drug development (PFDD). The most commonly used PRO measurements in the field of oncology include the Functional Assessment of Cancer Therapy-General (FACT-G), the European Organisation for Research and Treatment of Cancer Quality of Life Questionnaire (EORTC QLQ), and the Patient Reported Outcomes Measurement Information System (PROMIS) (Warsame and D’Souza, 2019).

### 2.4 Intercurrent event

The intercurrent event (ICE), e.g., discontinuation of assigned treatment, use of an additional or alternative treatment, and terminal events such as death, is one of the most central parts that ICH E9 (R1) emphasized. Envisioning and handling ICEs appropriately is important to precisely describe the treatment effect. However, there is often neglect in handling ICEs in early-phase clinical trials, which may result in intractable missing data for some key efficacy or safety outcomes. This is mainly because the uncertainty of the estimated treatment effect resulting from a small sample size is large, even if the handling of various ICEs is fully considered. On the other hand, the time required to collect the primary endpoints is shorter and the management of patients is more stringent in early-phase clinical trials. Thus, the impacts of ICEs on estimating treatment effects are not as large as those in Phase III confirmatory trials. There are now limited studies that provide strategies for handling ICEs in dose-schedule optimization trials. The focus of this paper is not on ICEs as well. We just want to make the readers rethink about the issue through the brief introduction in this section.

### 2.5 Population-level summary

In a dose-schedule optimization trial, population-level summaries that evaluate endpoints of interest should be pre-specified. For example, the primary population-level summary can be the probability of DLT in a phase I MTD-finding trial, while for some phase I-II trials focusing on both safety and efficacy, the summaries of tumor response or changes in biomarkers are also important. In recently developed Bayesian phase I-II clinical trial designs, the population-level summary can be the utility, a measurement of the toxicity-efficacy tradeoff (Zhou et al., 2019; Lin et al., 2020a). The population-level summary should reflect the goal of optimization and be placed at the center of the dose-schedule optimization process.

## 3 Bayesian adaptive designs for dose-schedule optimization

The trial design is a connection of those critical factors introduced in Section 2. It determines the process of a clinical trial and whether the trial can achieve its intended objectives. As clinical trials are conducted to address specific medical questions with limited resources, prospectively designing trials with adaptive features may allow more resources to be devoted to the best use. For example, the response adaptive randomization can assign more patients to dose-schedule regimens with better benefit-risk tradeoff and thus may improve the probability of identifying the optimal dosage. In this section, we review Bayesian adaptive designs for dose-schedule optimization. The assumptions, characteristics, limitations, and application scenarios of those designs are introduced, and the summary table of these designs (Supplementary Table S1) can be found in the Supplementary Material. We summarize the pros and cons of Bayesian methods, and some key elements of trial designs are discussed at the end of this section.

### 3.1 Dose-schedule optimization focusing on toxicity

At first, dose-schedule optimization designs were proposed mainly to deal with chronic and cumulative toxicities. Braun et al. (Braun et al., 2005) proposed a Bayesian adaptive design to determine a MTS rather than an MTD. The model is based on time-to-toxicity data, with the hazard of toxicity modeled as the sum of a sequence of ‘up-and-down’ triangular hazards, each associated with one administration. The candidate schedules are nested and the schedules assigned to newly enrolled patients are adaptively updated based on accumulated data. This design can incorporate the actual timing of individuals’ administrations, but it assumes only a single dose is under study. After that, Liu and Braun (Liu and Braun, 2009) proposed a phase I clinical trial design to find the MTS, based on a parametric non-mixture cure model. The hazard for each administration is proportional to a Weibull density, which is more flexible than the previously proposed triangular hazard. However, this method also assumes that there is only one investigational dose.

To address this issue, Braun et al. (Braun et al., 2007), Zhang and Braun (Zhang and Braun, 2013) further generalized the work of (Braun et al., 2005) respectively and developed Bayesian designs that can simultaneously optimize dose and schedule by allowing different hazards for each dose. The goal is to determine a maximum tolerated dose and schedule (MTDS). Zhang and Braun (Zhang and Braun, 2013) also considered optimizing the dose and schedule assignments within patients. This method can reevaluate the current assignment of each enrolled patient and automatically determines whether intrapatient dose-schedule reassignment is needed.

Different from the assumption of nested schedules in the above designs, Wages et al. (Wages et al., 2014) proposed a dose-schedule finding design that can be applied to both completely and partially ordered schedules. This design focuses on binary toxicity outcomes and is an extension of the partial order continual reassessment method (POCRM) (Wages et al., 2011). Compared with the designs introduced before, this method is simpler and may be more easily understood by clinicians.

### 3.2 Dose-schedule optimization considering both toxicity and efficacy

Most designs for dose-schedule optimization consider both toxicity and efficacy. Li et al. (Li et al., 2008) proposed a joint model for the probabilities of toxicity and efficacy, and apply a Bayesian isotonic transformation to make the estimated toxicity probabilities adhere to a pre-specified ordering. Then, with the order-constrained toxicity probabilities and the unordered efficacy probabilities, the design sequentially assigns patients to the optimal dose-schedule regimen, which has the maximal posterior probability that the toxicity probability is smaller than or equal to the physician-specified upper limit for toxicity and the efficacy probability is larger than or equal to the physician-specified lower limit for efficacy. Thall et al. (Thall et al., 2013) used joint utilities of time-to-toxicity and time-to-response to guide the dose-schedule optimization. They assumed non-nested schedules and adopted an adaptive randomization strategy to assign patients. Guo et al. (Guo et al., 2016) proposed a Bayesian dynamic model for a trinary patient outcome (no efficacy and no toxicity, efficacy and no toxicity, toxicity) to model the joint effects of dose and schedule. There is no need to assume whether the schedules are nested or non-nested, and the proposed model allows to borrow strength across dose-schedule regimens adaptively.

In the context of therapeutic cancer vaccines, Cunanan and Koopmeiners (Cunanan and Koopmeiners, 2017) proposed a two-stage, randomized Bayesian adaptive trial design to select the best vaccination schedule from several non-nested schedules, assuming the same dose levels. In stage 1, acceptable schedules are identified by pre-specified criteria, and the optimal schedule is selected based on the magnitudes of the immune response. If stage 1 does not give a conclusive result, the trial would continue to stage 2 and predictive probabilities are calculated to determine the sample size required for stage 2.

To address the issue of optimizing dose-schedule regimens within multiple disease subgroups, Quintana et al. (Quintana et al., 2016) proposed a Bayesian adaptive design for an adoptive T cell therapy. Safety data (i.e., DLT) for different subtypes is pooled while efficacy information (i.e., complete response) is borrowed across subtypes using a hierarchical dose-response model. Disease-specific utilities are used to guide dosage optimization, and an adaptive randomization approach is applied to dynamically assign patients. Lin et al. (Lin et al., 2020b) proposed a more widely applicable trial design that can handle delayed outcomes using likelihood-based approaches. Ordered disease subgroups and non-nested schedules are assumed. Utilities are used to quantify the efficacy-toxicity trade-off, and adaptive randomization is used to assign patients to candidate dose-schedule regimens. This method also allows information borrowing across subgroups, doses, and schedules. Shortly afterward, Lin et al. (Lin et al., 2021) further extended their previous work (Lin et al., 2020b) and relaxed the assumption of ordered disease subgroups. They also considered using more of the available data, including bioactivity and low-grade toxicity data, to predict the unobserved delayed outcomes.

### 3.3 Dose-schedule optimization incorporating PK/PD information

In nearly all early-phase clinical trials for oncologic diseases, PK and PD data would be collected and analyzed. But few studies have explicitly incorporated them into the process of dose-schedule optimization. Ursino et al. (Ursino et al., 2017) compared several methods that incorporate PK measurements in phase I dose-finding trials. They found that, although it does not improve the efficiency of dose-finding trials, adding PK measurements does allow better estimation of dose-toxicity curves. Günhan et al. (Günhan et al., 2020) proposed using the pseudo-PK model to describe the time-varying drug exposures and modeling the time-to-toxicity variable with the drug exposures. However, the PK data are not actual drug concentration data and the generation of pseudo-PK data needs support from previous PK studies, which may limit the practical application.

Gerard et al. (Gerard et al., 2021; Gerard et al., 2022) proposed Bayesian dose regimen assessment methods using PK/PD information to identify the maximum tolerated regimen at the end of a dose-escalation trial. The regimen-PK/PD model and PK/PD-toxicity model are integrated. They concluded that the inclusion of PK/PD information can help more precisely estimate the dose regimen toxicity and the methods they proposed may recommend alternative untested regimens for further study. These methods are *post hoc* analyses and therefore not a kind of trial design. With the development of quantitative pharmacology, we believe that more and more dose-schedule optimization studies will prospectively incorporate PK/PD information during dosage allocation.

### 3.4 Dose-schedule optimization for drug combination

As the drugs may have overlapping toxicities and can become intolerable when used in combination, additional dose-schedule optimization trials are needed. To the best of our knowledge, few studies have focused on this issue so far. Mozgunov and Jaki (Mozgunov and Jaki, 2019) simplified the complex dose-combination-schedule, and directly modeled efficacy and toxicity with candidate regimens. The regimen optimization can be achieved without any parametric or monotonicity assumptions. Similarly, Abbas et al. (Abbas et al., 2020) and Mozgunov et al. (Mozgunov et al., 2022) extended the POCRM and tailored it to adapt dose-schedule optimization for drug combinations respectively. However, it may sometimes be necessary to generate evidence regarding the contribution of each component in the drug combination. Those methods introduced above, although easy to implement, are more like single-agent dose-finding designs without monotonicity assumption to some extent.

### 3.5 Critique

**FIGURE 2:**
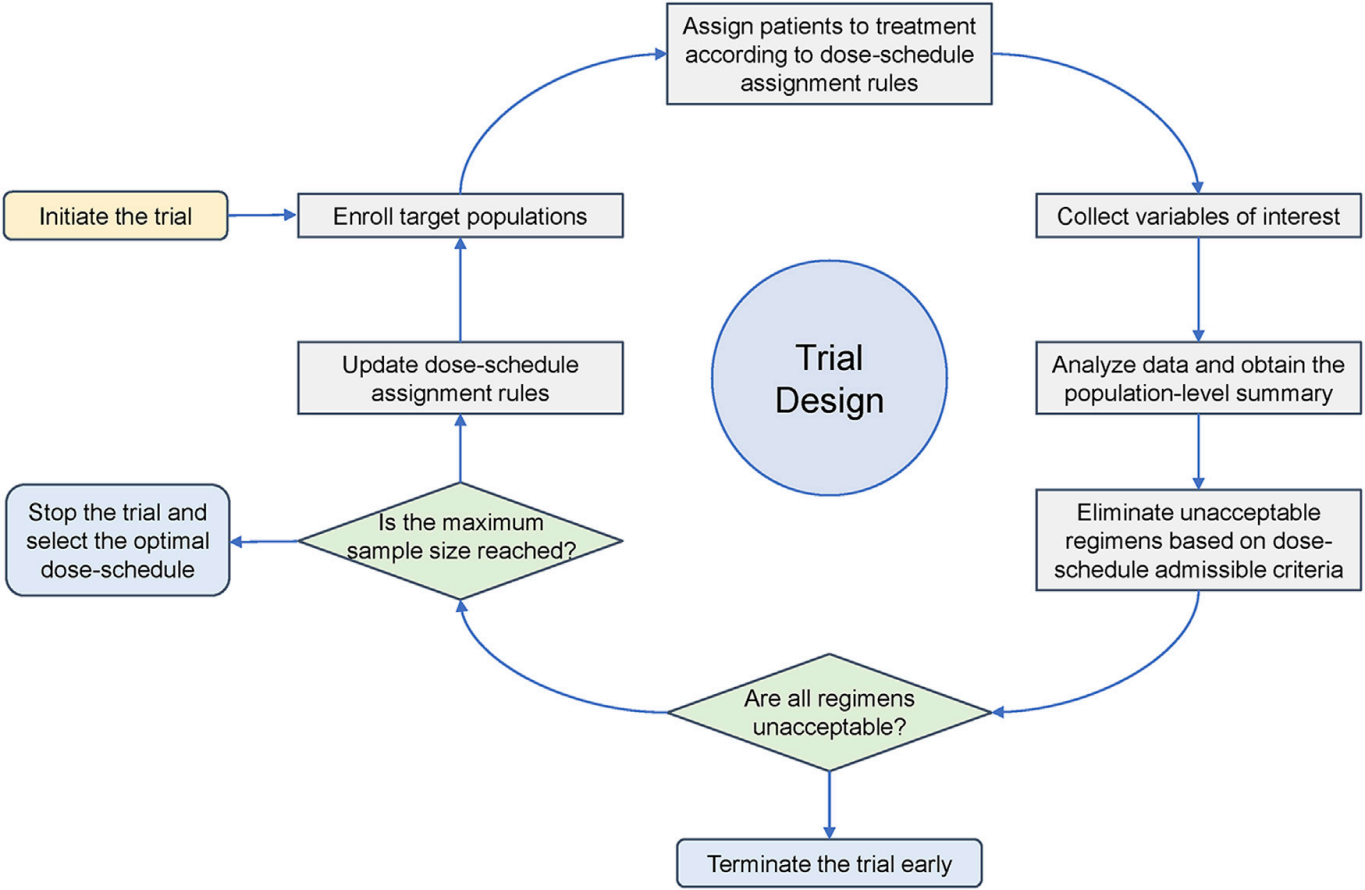
The main components of an adaptive dose-schedule optimization trial design.

The main components of an adaptive dose-schedule optimization trial design are summarized in Figure 2. Most of them, such as target population, variables of interest and population-level summary, have been introduced in Section 2. The dose-schedule admissible criteria, which is considered in almost all published designs, is set to stop enrollment to those futile or overly toxic dosages promptly. Then, let us turn our attention to the last, yet also very important, component that has not been discussed, the dose-schedule assignment rules. Typically, if the toxicity ordering of candidate dose-schedule regimens is completely or partially known, there is a tendency to assign patients sequentially to regimens in a non-randomized manner, like a dose-escalation trial. This avoids exposing patients to higher toxicity risks when uncertainties about safety are still high. In contrast, if the toxicity ordering of candidate regimens is unknown or the safety uncertainties are controllable, patients are often assigned randomly to admissible regimens. A randomized, parallel dose-response trial is recommended to compare dosages by FDA (FDA, 2023), as it ensures the similarity of patients receiving each dose-schedule regimen and interpretability of dose-response (including both dose-toxicity and dose-efficacy) relationships. Two practical implementation examples of the presented designs are summarized in Table 1 based on the essential design components in Figure 2.

**TABLE 1:** Summary of two practical implementation examples of the presented designs.

Braun et al. Braun et al. (2007)	
Objective	To determine the maximum tolerated dose and schedule of Vidaza for patients with acute myelogenous leukemia (AML) who received allogeneic blood or bone marrow cell transplantation
Treatment	12 candidate dose-schedules of Vidaza
	Three doses: 8, 16 and 24 mg/m^2^
	Four nested schedules: 1, 2, 3 and 4 courses
Population	AML patients who received allogeneic blood or bone marrow cell transplantation
Variable	Time-to-toxicity
Population-level summay	Cumulative toxicity probability
Dose-schedule admissible criteria	The posterior probability that the toxicity probability of the dose-schedule higher than a fixed upper bound is smaller than a pre-specified cutoff
Dose-schedule allocation rules	Within the admissible dose-schedules, assign the next patient to the dose-schedule whose posterior mean cumulative toxicity probability is closest to the target toxicity probability
Mozgunov et al. Mozgunov et al. (2022)	
Objective	To determine the maximum tolerated dose-combination-schedule of niraparib plus M1774 for patients with metastatic or locally advanced unresectable solid tumors
Treatment	20 dose-combination-schedules of Niraparib plus M1774
	Two doses for Niraparib: 100 and 200 mg
	Five doses for M1774: 30, 60, 90, 130 and 180 mg
	Two nested schedules for M1774: continuous once daily and once daily with breaks that is approximately half as intensive as the first schedule
Population	Patients with metastatic or locally advanced unresectable solid tumors
Variable	DLT event
Population-level summay	DLT probability
Dose-schedule admissible criteria	The posterior probability that the DLT probability of the regimen higher than a fixed upper bound is smaller than a pre-specified cutoff
Dose-schedule allocation rules	Within the admissible regimens, assign the next cohort of patients to the regimen by a pre-specified criterion that takes into account both the uncertainty in toxicity estimates and penalization for overdosing

Why we emphasize Bayesian methods in this review lies in several aspects. First, Bayesian posterior probabilities, e.g., the probability that regimen A has a higher DLT rate than regimen B, are more intuitive and comprehensible than *p*-values, making it easier for clinicians to make informed decisions. Second, Bayesian methods can facilitate the synthesis of clinical evidence conveniently by introducing appropriate priors, which is attractive for early-phase clinical trials with small sample sizes. Third, the accumulated data from dosage optimization trials can continuously update the posteriors, which is consistent with the iterative ‘learn and confirm’ paradigm for drug discovery (Sheiner, 1997). For more about how Bayesian methods can be applied to benefit-risk assessment, the readers may refer to Costa et al. (Costa et al., 2017).

There are also some obstacles that hinder the application of Bayesian methods in optimizing dose-schedule regimens, the most prominent of which is the use of subjective prior beliefs. To date, no universally accepted methods for eliciting priors exist and sensitivity analyses are always needed. In addition, due to the fast pace and potentially seamless progression of oncology clinical trials, the analyses should be prioritized and performed in real-time fashion in order to timely assist decision-making (Ji et al., 2018). However, there are now few user-friendly desktop software available, as can be seen from Supplementary Table S1. In most cases, the trialists have to determine a lot of parameters when applying such Bayesian designs. It is recommended that clinicians first provide some options for clinically intuitive parameters, e.g., the lower limit of acceptable response rate. Then, the choices of parameters should be evaluated by simulation and calibration. Repeated discussions and modifications on the parameter choices can usually take several times. Programming, validating and conducting simulation studies can be more time-consuming for a Bayesian adaptive design. But in general, we still believe that the Bayesian methods will play a more important role in early-phase dose-schedule optimization trials.

It should be noted that all of the methods we have introduced before rely on some assumptions, such as the ordering of schedules, the ordering of subgroups, the exposure-response relationships, *etc.* If the assumptions are violated, the analysis results may be misleading and the identified dose-schedule regimen may not be optimal. The readers may now have a deeper appreciation of how trial designs can connect those factors introduced in Section 2, and it is recommended to carefully evaluate those five attributes before designing a dose-schedule optimization trial. It is also important to emphasize that those existing Bayesian adaptive designs, as well as some other conventional trial designs and MIDD strategies, are not mutually exclusive, and they can be integrated appropriately to align trial-specific objectives.

## 4 Opportunities

As illustrated in Section 3, the Bayesian adaptive design is a potentially effective way to evaluate several doses and schedules simultaneously in a single clinical trial with higher efficiency. Dozens of publications have proposed specific designs for different clinical scenarios. However, there are still some issues that have not been fully considered or have not been addressed. In Section 4 and Section 5, several future research opportunities and challenges are presented respectively for Bayesian dose-schedule optimization trial designs.

### 4.1 Patient-focused drug development (PFDD)

A prospective phase I patient survey concludes that adverse events (AEs) considered intolerable by patients are toxicities that directly impact their quality of life and differ from those feared by physicians or included in the DLT definition (Henon et al., 2017). Therefore, optimizing dose-schedule regimens should be patient-focused, ensuring that patients’ experiences, perspectives, needs, and priorities are captured (Schroeder et al., 2022). Clinical outcome assessments (COAs), especially PROs, are getting more and more attention in clinical research.

The ubiquity of smartphones makes it more convenient to collect PRO data. Compared with traditional assessments of safety and efficacy (e.g., laboratory testing and imaging examination), the frequency of PRO assessment can be higher, thus it can reflect the patient’s quality of life in a more timely manner. In the context of dose-schedule optimization, PRO data can be prediction signals for toxicity and efficacy, or be directly incorporated into the benefit-risk assessment. To the best of our knowledge, there are now limited dosage optimization designs considering PRO data explicitly. The opportunities for constructing Bayesian joint models and benefit-risk trade-off criteria, which link longitudinal PRO data with toxicity and efficacy endpoints, are immense.

### 4.2 Using external data

Utilizing information from external data, such as preclinical data, real-world data, or historical data from clinical trials, is one of the distinguishing features of Bayesian trial designs. For example, in the dose-escalation study of asciminib for patients with chronic myeloid leukemia, a Bayesian logistic regression model was used to estimate the DLT probabilities of various dose-schedule regimens (Hughes et al., 2019). It can be seen from the trial protocol that weakly informative priors were derived for model parameters based on pre-clinical and historical data.

In the past few years, several single-agent dose-finding designs have been proposed to borrow information from external data (Liu et al., 2015; Li and Yuan, 2020; Zhou et al., 2021; Chen et al., 2022; Lin et al., 2022). Hashizume et al. (Hashizume et al., 2023) further considered incorporating single-agent historical data into drug combination phase I cancer trials. It is expected that in the near future, researchers would propose more complex innovative Bayesian designs allowing borrowing strength from external data for dose-schedule optimization trials.

### 4.3 Model-informed drug development (MIDD)

A recent review of phase I immuno-oncology trials found that positive PD biomarker results were infrequently correlated with clinical activity or cited in subsequent trials (Salawu et al., 2022), suggesting that PK/PD information has not been fully considered in drug development. MIDD, commonly used to describe the application of a wide range of quantitative models in drug development to facilitate the decision-making process (Wang et al., 2019), has a huge potential to integrate PK/PD information in clinical studies. Well-known technologies for MIDD include PK/PD model, exposure-response (ER) model, population pharmacokinetic model (Pop-PK), physiologically based pharmacokinetic model (PB-PK), quantitative systems pharmacology (QSP), model-based meta-analysis (MBMA), and so forth. For example, publications (Gerard et al., 2021; Gerard et al., 2022) introduced in Section 3.3 use nonlinear mixed-effects models to link the PK data with the dose-schedule regimens, which is a typical approach of Pop-PK.

There are several real examples where MIDD approaches are applied for dose-schedule optimization, such as nivolumab, pembrolizumab and atezolizumab (Peer et al., 2020). The model-informed dose-schedule optimization is usually carried out when a clinical trial is completed. Then, the recommended dose-schedule regimen will be tested and confirmed in a new clinical trial. Considering that pre-specifying candidate dosages before a dose-optimization trial may be troublesome when the clinical data is limited, the authors believe that it is promising to incorporate MIDD approaches to adjust candidate dosages and guide the dose-schedule optimization in the course of the clinical trial.

## 5 Challenges

### 5.1 Novel anti-tumor therapies

Novel anti-tumor drug classes, e.g., bispecific antibody, therapeutic cancer vaccine, cellular therapy, and gene therapy, are rapidly emerging in recent years. Their MOAs can be entirely distinct from those of classical drugs, calling for new customized dosage optimization designs. For example, the efficacy and on-target toxicity of bispecific antibodies may be driven by trimer formation (complexes between the bispecific antibody, T cell and tumor cell), resulting in a bell-shaped exposure-response relationship (Betts and van der Graaf, 2020). Therefore, it can be more challenging to optimize dose-schedule regimens for bispecific antibodies. In this case, a maximum tolerated regimen is generally not an optimal regimen, and the strategy for optimizing dose-schedule regimens should be carefully considered. One potential way is to consider maximizing the concentration of trimer formation when designing a dose-schedule optimization trial.

Delayed outcomes, which are common occurrences in novel anti-tumor therapies (Paoletti et al., 2014; de Miguel and Calvo, 2020; Dromain et al., 2020), can result in the missingness of data when the interim analysis is to be conducted. Some designs, such as (Lin et al., 2020b; Lin et al., 2021), are proposed to deal with delayed outcomes in dose-schedule optimization trials. Apart from missing data, the assessment windows for toxicity and efficacy should also be determined carefully in the designing stage. The assessment window should be long enough to ensure the delayed outcomes can be identified, but a too long assessment window could inevitably prolong the trial duration and increase the probability of dropout. Close collaboration between clinicians and statisticians is required to determine an appropriate assessment window.

Some designs introduced in Section 3 were proposed for specific therapies, such as the therapeutic cancer vaccine (Cunanan and Koopmeiners, 2017) and cellular therapy (Quintana et al., 2016). However, those designs are not enough to meet the requirements of optimizing dose-schedule regimens for all novel therapies, and new treatments are springing up like mushrooms. How to design tailored dose-schedule optimization trials according to the characteristics of the investigational drugs is still a major challenge for clinical trialists.

### 5.2 Complex endpoints

Assessing benefit-risk trade-offs and optimizing dose-schedule regimens often involve complex endpoints. For example, lower-grade but persistent symptomatic toxicities are often concerns in immunotherapy. Several approaches have been proposed to account for multiple toxicity grades by assigning severity weights to each grade and type of toxicity event (Bekele and Thall, 2004; Yuan et al., 2007; Lee et al., 2012; Mu et al., 2019), but the elicitation of severity weights can be challenging for both biostatisticians and physicians. In terms of PRO endpoints, the types of response options can be a Likert scale, a rating scale, or a visual analog scale. This may bring challenges for data analysis and interpretation, especially when PRO endpoints are integrated with other types of endpoints. In addition, joint evaluations of safety, efficacy and PK/PD endpoints, which may include a mixture of continuous and discrete variables, also make the issue of complex endpoints more prominent. It is indeed necessary to develop innovative Bayesian dose-schedule optimization designs for complex endpoints.

### 5.3 Limited sample size and heterogeneous populations

Compared to cytotoxic chemotherapies, the target population of a targeted therapy may be much smaller, leading to more difficult recruitment of patients. However, dose-schedule optimization usually requires a higher sample size than traditional dose-escalation trials, as the number of candidate regimens may increase and the optimization may not just focus on toxicity. A rule of thumb in traditional phase I dose-escalation trials is that the maximum sample size is six times the number of pre-specified doses, but this may be insufficient for dose-schedule optimization. Sometimes it is necessary to compare the benefit-risk ratios between regimens, further increasing the demand for sample sizes. Therefore, when selecting the optimal dose-schedule regimen, the uncertainties resulting from limited sample sizes can be large. Although a dose-schedule optimization trial does not necessarily need to be powered to determine statistical superiority, it should be designed to detect early efficacy and safety signals and identify recommended dosages for subsequent studies. Aiming at this issue, it is recommended to fully utilize external data and identify potential adaptive modifications in the planning stage of clinical trials. Another practical issue related with the sample size is the trial duration, which is difficult to estimate. The trial duration depends on the sample size, the assessment window, the accrual rate and the algorithm for regimen assignments. Extensive simulation studies should be conducted to help determine the required sample size and estimate the trial duration.

Another challenge for dose-schedule optimization is the population heterogeneity. For example, the recommended dosages for several targeted therapies, e.g., trastuzumab deruxtecan (Shitara et al., 2019; Tamura et al., 2019) and asciminib (Hughes et al., 2019; Rea et al., 2021), are optimized to be population-specific. Some published designs like (Quintana et al., 2016; Lin et al., 2021), make it possible to optimize dose-schedule regimens for multiple subpopulations simultaneously in a single trial. However, although the strategy for borrowing information across subpopulations can improve the efficiency of clinical trials, it may also cause biased estimates and erroneous decision-making. It is recommended to carry out extensive simulation studies to quantify both favorable and unfavorable effects of information borrowing.

## 6 Conclusion

The recent development of novel anti-tumor treatments and combination therapies results in an increasing recognition of the essential place of optimizing dose-schedule regimens. This review summarizes critical factors associated with dose-schedule optimization from the perspective of the estimand framework. Then, related innovative Bayesian adaptive designs are reviewed and some comments about the pros, cons, and matters that need attention when adopting these designs are given. At last, we summarize some future research opportunities and challenges for dose-schedule optimization. The authors hope that this review can help clinical trialists consider various issues holistically when designing a dose-schedule optimization trial. We also expect physicians, biostatisticians, pharmacologists, and other stakeholders to work together and develop more effective tools for dose-schedule optimization.
