# Supplementary material for: Optimizing dose-schedule regimens with bayesian adaptive designs: opportunities and challenges
Source: Front Pharmacol. 2023 Nov 23;14:1261312. doi: 10.3389/fphar.2023.1261312 (PMC10701540; doi:10.3389/fphar.2023.1261312)
Supplement: Supplementary file 1 [file DataSheet1.PDF]

Supplementary Material

Optimizing Dose-Schedule Regimens with Bayesian Adaptive Designs: Opportunities and Challenges

Xin Chen[1]†, Ruyue He[1]†, Xinyi Chen[1]†, Liyun Jiang[1*], Fei Wang[1*]

[1] Research Center of Biostatistics and Computational Pharmacy, China Pharmaceutical University, Nanjing, China

† These authors contributed equally to this work and share first authorship.

*** Correspondence:**
Liyun Jiang
ljiang.cpu@foxmail.com

Fei Wang
wangfei@cpu.edu.cn

1 Supplementary Table

Supplementary Table 1. Summary of Bayesian adaptive designs for dose-schedule optimization.

Design	Assumptions	Application scenarios	Endpoints	Advantages	Limitations	Software
Braun et al. (19)	Nested schedules	MTS finding	Time-to-toxicity	It accounts for a patient's entire sequence of administrations.	The dose in each administration is fixed. The effects of multiple administrations are assumed to be independent.	Code is available by request.
Liu and Braun (20)	Nested schedules	MTS finding	Time-to-toxicity	It has more direct interpretations for model parameters than Braun et al (19).	The dose in each administration is fixed. The effects of multiple administrations are assumed to be independent.	Not mentioned
Braun et al. (21)	Nested schedules	MTDS finding	Time-to-toxicity	Dose and schedule can be optimized simultaneously.	The effects of multiple administrations are assumed to be independent.	Not mentioned
Zhang and Braun (22)	Nested schedules	MTDS finding	Time-to-toxicity	It allows optimizing intrapatient dose-schedule assignment.	In a study with faster accrual, patients could have several dose-schedule reassignment, which is impractical.	Code is available by request.

Design	Assumptions	Application scenarios	Endpoints	Advantages	Limitations	Software
Wages et al. (23)	Partially ordered schedules	MTDS finding	Toxicity probability	As an extension of CRM, it is more easily understood by clinicians.	The number of all possible dose-schedule orderings may be large, and the subset of orderings should be chosen prudently.	http://faculty.virginia.edu/model-based_dose-finding/
Li et al. (25)	Schedules are nested for toxicity and non-nested for efficacy	Find the optimal dose-schedule with tolerable toxicity and high efficacy	Toxicity and efficacy probability	The order constraint between schedules is dealt with by isotonic transformation.	It may not be appropriate in the presence of late onset toxicity or efficacy.	Not mentioned
Thall et al. (26)	Non-nested schedules	Find the optimal dose-schedule based on utility	Time-to-toxicity and time-to-response	Adaptive randomization is used to avoid getting stuck at a suboptimal regimen.	The utility functions and distributions of event time are not easy to specify.	Not mentioned
Guo et al. (27)	Non-nested schedules	Find the optimal dose-schedule that is safe and has the highest efficacy	Trinary outcomes: no efficacy and no toxicity, efficacy and no toxicity, toxicity	It allows borrowing strength across dose-schedule regimens.	It may not be appropriate in the presence of late onset toxicity or efficacy.	http://wileyonlinelibrary.com/journal/rss-datasets
Cunanan and Koopmeiners (28)	Non-nested schedules	Find the optimal schedule based on toxicity and immune response	Toxicity probability and immune response	The sample size of the second stage is determined based on the results from the first stage.	The dose level is the same for candidate schedules. Immune response may be an inexact surrogate for clinical response.	Not mentioned
Quintana et al. (29)	Nested schedules	Find the optimal dose-schedule for multiple disease subgroups	Toxicity and efficacy probability	It allows borrowing efficacy information across disease subgroups. The utility function for optimization is disease-specific.	The operating characteristic is sensitive to the prior distributions and probability thresholds, and the calibration may be complicated.	Not mentioned

Design	Assumptions	Application scenarios	Endpoints	Advantages	Limitations	Software
Lin et al. (30)	Non-nested schedules	Find the optimal dose-schedule for ordered disease subgroups	Toxicity and efficacy probability	It allows borrowing strength across dose-schedule regimens and disease subgroups. Delayed outcomes can be handled.	When there is a mixture of homogeneous and heterogeneous subgroups, Bayesian hierarchical model may lead to incorrect decisions.	The R code for is available in the literature.
Lin et al. (31)	Non-nested schedules	Find the optimal dose-schedule for unordered disease subgroups	Toxicity and efficacy probability	It allows borrowing strength across dose-schedule regimens and disease subgroups. Delayed outcomes can be imputed by auxiliary information.	When there is a mixture of homogeneous and heterogeneous subgroups, Bayesian hierarchical model may lead to incorrect decisions.	Code is available by request.
Günhan et al. (33)	Nested schedules	MTDS finding	Time-to-toxicity	By incorporating the PK information, the sample size required may be lower.	The generation of pseudo-PK data needs support from previous PK studies.	https://github.com/gunhanb/TITEPK_code
Mozgunov and Jaki (36)	Partially ordered dose-combination schedules	Find the optimal combination-schedule	Trinary outcomes: no efficacy and no toxicity, efficacy and no toxicity, toxicity	It does not need any parametric or monotonicity assumptions.	The information theoretic method is not easily understood by clinicians.	Not mentioned
Abbas et al. (37)	Partially ordered dose-combination schedules	Find the maximum tolerated combination-schedule	Toxicity probability	It is easy to implement.	The number of all possible dose-combination-schedule orderings may be large, and the subset of orderings should be chosen prudently.	https://github.com/dose-finding/comparison-non-mono

Abbreviation: MTS, maximum tolerated schedule; MTDS, maximum tolerated dose and schedule; CRM, continual reassessment method; PK, pharmacokinetic.
